# A New Era of National Guideline Development in Saudi Arabia

**DOI:** 10.1007/s44197-022-00076-y

**Published:** 2022-11-28

**Authors:** Ziad A. Memish, Abdulrahman S. Alqahtani, Nahar Al-Azemi, Nebras Abu Alhamayel, Mohammad Saeedi, Shatha Abuzinada, Rayan G Albarakati, Subramaniasivam Natarajan, Ximena Alvira, Khushnam Bilimoria, Klara Brunnhuber

**Affiliations:** 1 Research & Innovation Centre, King Saud Medical City, Ministry of Health & College of Medicine, AlFaisal University, Takhassusi St, P.O. Box 50927, Riyadh, 11533 Kingdom of Saudi Arabia; 2 Lead National Guideline Center, Health Holding Company, Ministry of Health, Riyadh, Kingdom of Saudi Arabia; 3 Hubert Department of Global Health, Emory University, Atlanta, GA GA30322 USA; 4 Transformation Sector, Health Holding Company, Riyadh, Kingdom of Saudi Arabia; 5 Saudi Health Council, Riyadh, Kingdom of Saudi Arabia; 6 Strategic Design and Innovation, Model of Care-Center of Excellence, Riyadh, Kingdom of Saudi Arabia; 7 National Center for Evidence-Based Medicine, Saudi Health Council, Riyadh, Kingdom of Saudi Arabia; 8 Department of Obstetrics and Gynecology, Majmaah University, AL-MAJMAAH, 11952 Kingdom of Saudi Arabia; 9 Clinical Solutions, Elsevier España S.L.U, Barcelona, Spain; 10 Clinical Solutions, RELX India Pvt. Ltd, Gurugram, Haryana India; 11 Clinical Solutions, Elsevier Ltd, London, UK

**Keywords:** Clinical practice guidelines, Health Sector Transformation, Value-based care, Patient outcomes, Evidence-based medicine, GRADE Methodology, Electronic order sets, Guidelines International Network

## Abstract

Saudi Arabia’s ambitious Vision 2030 project was launched in 2016 as a strategy for economic development and national growth, with 11 Vision Realization Programs put in charge of its implementation. The backbone of its Transformation Program for the Health Sector has been the definition of a new Model of Care aiming to deliver 42 coordinated interventions across 6 Systems of Care, with the development of clinical guidelines identified as a key cross-cutting intervention to foster the use of national, evidence-based practices across KSA, reduce care variation, and promote accountable care. This article provides an overview of the history, progress to date, and future outlook of the recently initiated National Guidelines Center in Saudi Arabia, established in collaboration between the Health Holding Company and the Saudi Health Council represented by its National Center for Evidence-based Medicine. The lessons learnt from previous guideline initiatives are grouped under the Center’s design principles of high quality, relevance, practical implementation, and sustainability. Aspects setting the project apart from previous endeavors have been its focus on extensive engagement with key stakeholders in the Saudi guideline ecosystem, the co-development of evidence-based recommendations with aligned key performance measures, and the implementation of guideline recommendations in the clinical workflow via integrated electronic order sets. Nine activity streams aim to enable the Center to take its place among the leading regional and global guideline developing organizations and to optimally support clinicians and patients, Saudi Arabia’s health sector transformation, and the work of guideline communities worldwide.

## Introduction

Saudi Arabia (KSA) has one of the highest healthcare expenditures in Gulf Cooperation Council countries (GCC), as well as one of the highest densities of medical professionals per thousand residents [1]. This allows KSA Ministry of Health to provide wide health coverage to both Saudi citizens and expatriates through its widely distributed network of hospitals and primary healthcare centers. Health services in Saudi Arabia (KSA) are provided free of charge to all Saudi citizens and expatriates working in the public sector. All expatriates’ workers living in KSA are required to have private health insurance covered by their employers. Health services for this population are provided through the rapidly flourishing private sector.

However, non-communicable diseases, particularly diabetes and obesity, continue to burden the kingdom, and are a major target of preventive care screening policies. It has been estimated that the obese population will reach 73% by 2025 [1]. Conversely, communicable diseases, particularly maternal, and neonatal-associated diseases, have observed a steep decline according to data from 2019 compared to 1990 [2].

In response to this situation in 2016, Saudi Arabia launched the ambitious Vision 2030 project as a strategy for economic development and national growth. Eleven Vision Realization Programs (VRPs) have been put in charge of its implementation, including a dedicated VRP and Transformation Program for the Health Sector [3, 4].

**Fig. 1:**
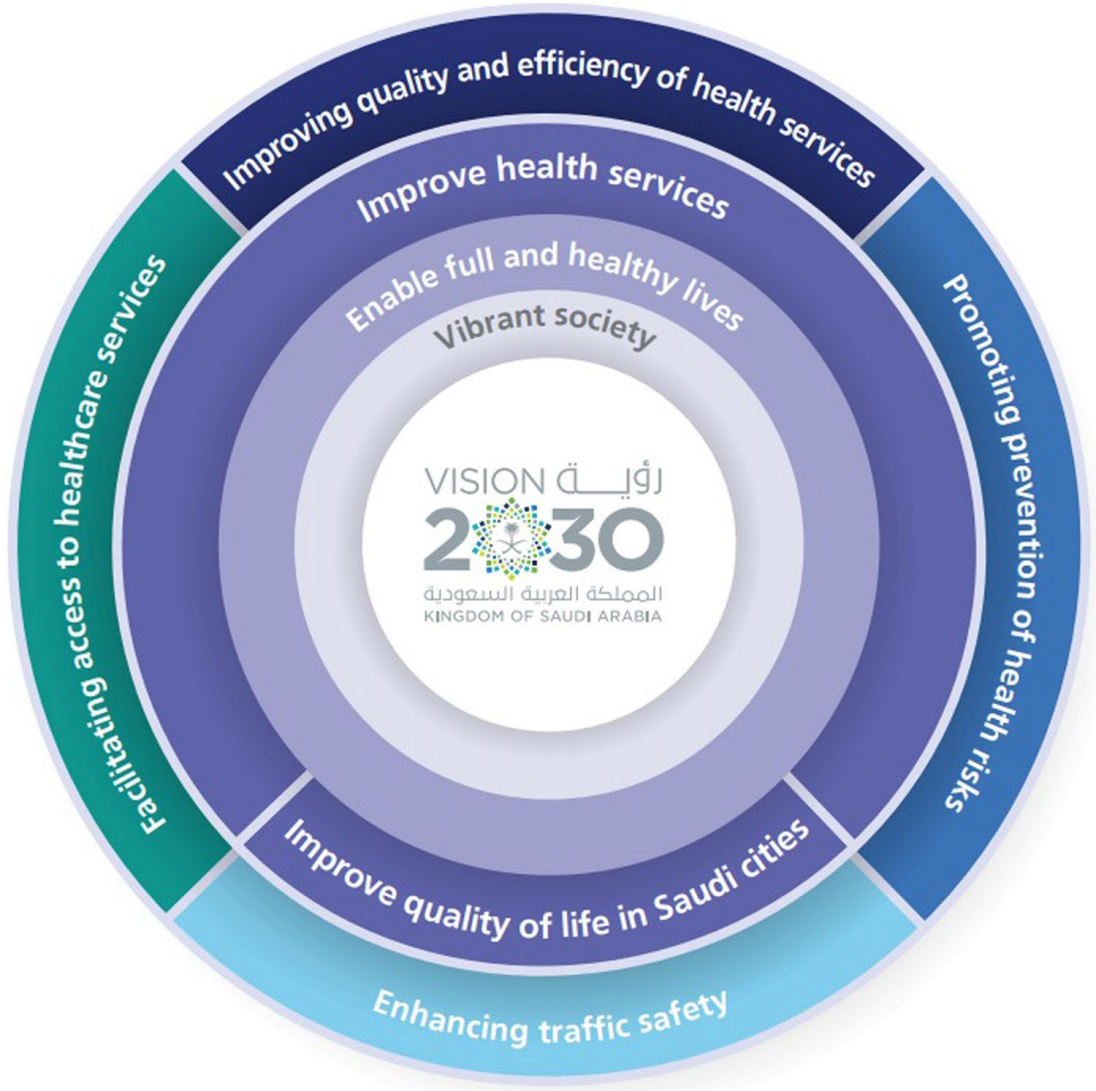
Strategic goals for health transformation in Kingdom of Saudi Arabia

The backbone to healthcare transformation is the definition of a new Model of Care that, among other objectives, aims to deliver 42 coordinated interventions across 6 Systems of Care (Keeping well; Planned procedure; Women & children; Urgent problems; Chronic conditions; and Last phase of life) responsible for the delivery of all health services (Fig. 1) [5]. The development of clinical guidelines had been identified as a key cross-cutting intervention.

Key to achieving the healthcare transformation within the kingdom’s Vision 2030 program was to promote the use of national, evidence-based practices across KSA to reduce care variation and promote accountable care, through regular adaptation/development, alignment, training, and dissemination of localized clinical practice guidelines.

In close collaboration with the Saudi Health Council (SHC) represented by its National Center for Evidence-based Medicine, the Health Holding Company (HHC)—established by the Saudi Ministry of Health to drive its Model of Care Health Sector Transformation strategy—has initiated a National Guidelines Center to guide clinical practice and be the advocate for evidence-based medicine, thereby improving the wellbeing of its nation in a sustainable, standardized, and accountable manner.

The SHC was established by a Royal Decree issued in June 2002, with its council under the chairmanship of the Minister of Health and whose current members include all health care providing sectors in the kingdom like Ministry of Health, Military hospitals, Saudi Red Crescent Authority and the special regulatory entities i.e Saudi Food and Drug Authority (SFDA), Council of Collaborative Health Insurance (CCHI ) and Saudi Commission for Health Specialties (SCHS) and other ministries like the Ministry of Economy and Planning, the Ministry of Labor and Social Development and the Ministry of Finance. Key among its many tasks of the SHC was to prepare the healthcare strategy in KSA, issue appropriate regulations to ensure that hospitals run by the Ministry of Health and other government agencies are operated in adherence to the principles of economic management as well as performance and quality standards and development and approval of policies for the coordination and integration between all healthcare providing authorities.

Over the last few decades, numerous organizations across Saudi Arabia have independently undertaken the task to create documents aimed at guiding clinical decisions, such as guidelines, consensus documents, pathways, and protocols [6]. Despite the dedication and hard work of the involved experts, these largely disjointed efforts have been hampered by numerous factors, which have resulted in a lack of reliable, clinically credible, accessible, locally applicable, and nationally adopted guidelines, ultimately impacting on the overall healthcare objectives of the Saudi Vision 2030 project. In response, HHC and SHC developed a strategic plan to address these limitations aligned with the six guiding principles for the project: (i) promote evidence-based medicine; (ii) drive value-based care; (iii) enhance patient and health outcomes; (iv) leverage local and international data-driven clinical guidance; (v) operate in an inclusive and collaborative manner within the ecosystem; and (vi) focus on both curative and preventive health.

Table 1 lists the lessons learnt from previous guideline initiatives, reflecting the Center’s design principles of high quality, relevance, practical implementation, and sustainability.

**Table 1:** Transferrable lessons learnt from Saudi guideline projects to date grouped by the National Guidelines Center’s four design principles for guideline development

Lessons learnt	Solution implemented at the National Guidelines Center in Saudi Arabia
Design principle 1: high quality	
Ensure comprehensive conflict of interest declaration	All clinical experts involved in voting sessions (in addition to all people involved in any part of guideline development) declare their COI at the start of the process in accordance with the Guideline Center’s COI policy based on GIN’s 9 Guiding Principles [8]
Apply rigorous evidence-based methodology	Guidelines are developed using methodologies developed by the GRADE Working Group and GIN (such as the Adolopment methodology and Evidence to Decision frameworks developed by the GRADE Working Group) [9–11] Guidelines are developed in partnerships with companies with known experience in creating methodologically rigorous international guidelines (for example, Elsevier [https://www.elsevier.com/] and Epistemonikos Foundation [https://www.epistemonikos.cl/]) All active Task Force (expert panel) members are given the opportunity to complete a INGUIDE Level 1 Guideline Group or Panel Member Certification Course jointly developed by GIN and McMaster University’s Department of Health Research Methods, Evidence, and Impact (https://inguide.org/)
Design principle 2: relevance	
Involve key stakeholder groups	In line with global best practice, stakeholder buy-in and ownership has been sought from the earliest stage of the project via extensive stakeholder consultation in the form of 3 workshops, 2 surveys, and 10 post-survey interviews for alignment with over 60 key stakeholders during formulation of foundational Guidelines Center documents, policies and processes (Vision, Mission, Charter, Design principles, Guideline Development Processes, Conflict of Interest Policies, Governance Structure, Guideline Topic Selection etc.) [12] End users and representatives of key stakeholder organizations involved in the healthcare process or guideline implementation are invited to participate in peer review
Utilize local expertise	Guideline development is undertaken (with the help of a methodology and administrative support team) by multidisciplinary panels (“Task Forces”) of around ten people each, comprised of local healthcare professionals across the specialties relevant to the guideline The selection of clinical questions by guideline task forces is informed by local clinical priorities and needs for each topic and usually covers several settings and/or areas across the care continuum such as prevention, diagnosis, treatment, discharge, and follow-up
Focus on local needs and value	The selection and prioritization of guideline topics is based a multi-component framework comprised of guideline impact indicators (such as local or regional epidemiology and disease burden) and effort parameters (e.g. availability and maturity of local care pathways, national guideline centers or teams) Value is built into the very fabric of the project and the Center, for example through Close involvement of the Center for Improving Value in Health (https://cvalue.sa) Input on topic selection and other strategic decisions from insurer and payer organizations Systematic consideration of cost as one of the contextual factors when formulating guideline recommendations
Design principle 3: practical implementation	
Develop a varied and timely dissemination and implementation strategy	The guidelines produced by the Guidelines Center will be disseminated to end users through online and offline channels including a website, mobile apps, publications, and educational events Order sets in electronic patient record systems at selected pilot sites are being aligned with the new national guidelines, enabling automated monitoring of clinical adoption and impact Key performance indicators are being co-developed for selected recommendations to provide starting points for local audits and quality improvement initiatives to drive guideline use and adherence
Design principle 4: sustainability	
Identify the optimal long-term host for the National Guidelines Center	HHC has conducted interviews with 18 stakeholder organizations across the Saudi guideline landscape to establish the most effective future governance model for the Center that resulted in consensus for a National Guidelines Center to move to the SHC and its National Center for EBM
Spread the workload	To ensure methodological consistency across all guideline developing organizations in Saudi Arabia, the SHC National Center for EBM has developed a booklet outlining the key principles of guideline development based on the GIN standards and aligned with the methodology used for the first 12 guidelines developed by the Center [5]
Build strategic partnerships and collaborations	Entering the next phase of the Center under the aegis of the SHC, one key focus will be on (re-)establishing good working relationships with local guideline expert methodologists and earlier local guideline initiatives inside Saudi Arabia (e.g. with the Guideline Adaptation Program at the King Saud University, National Gulf EBM Center in the National Guard, Jeddah EBM Group, BORHAN [Saudi Society for EBHC], the Saudi Commission for Health Specialties) The Center is keen to engage in dialogue with guideline centers worldwide for continuous exchange, learning and improvement about best practices in guideline development and implementation, with several sites visits in planning Once fully functional, the Center will aim to become a hub or resource/reference center at regional and international levels for collaborations and networking in the areas of guideline development, evidence-based healthcare, and knowledge translation projects (e.g. with WHO-EMRO, WHO Collaborating Centers, GIN, Arab Regional Community, International Society for Evidence-Based Health Care [ISEHC], the GRADE Working Group, JBI, Cochrane)
Utilize digital tooling for transparent documentation and audit	HHC and SHC are using advanced digital tooling to support and document various steps of guideline development, to facilitate participation of the Task Force members, and to ensure ease of review and updating of all captured information and data
Ensure regular and timely guideline updates	The timing of guideline review (and if required, updating) will be informed by continuous evidence scanning with a guideline expiry date 5 years following publication

*COI* Conflicts of Interest, *EBM* Evidence-Based Medicine, *GIN* Guidelines International Network, *GRADE* Grading of Recommendations Assessment, Development and Evaluation, *HHC* Health Holding Company, *SHC* Saudi Health Council

Important aspects setting our project apart from previous endeavors and aiming to inform similar endeavors worldwide include its focus on extensive engagement with key stakeholders in the Saudi guideline ecosystem, the co-development of evidence-based recommendations with aligned key performance measures, and the implementation of national guideline recommendations in the clinical workflow at the point of care via integrated electronic order sets.

## Project Progress to Date

As of October 2022, several milestones have been reached including: (i) documentation of the Center’s vision and mission, charter, goals, processes, and policies; (ii) extensive stakeholder engagement with organizations active in the Saudi guideline ecosystem; (iii) confirmation of the SHC National Center for Evidence-Based Medicine as the most suitable entity to host the center following consultation among 19 stakeholder organizations using a RACI (Responsible/Accountable/Consulted/Informed) framework; (iv) based on a data-driven impact/effort analysis, selection of the initial 12 guideline topics as follows: Chronic kidney disease, Dental caries, Community-acquired pneumonia, Cesarean section, Stroke, Sepsis, Acute gastroenteritis in children, Low back pain in adults, Mechanical ventilation, Chronic obstructive pulmonary disease, and Depression; (v) Recruitment of Clinical Leads and multidisciplinary Task Forces for all topics; and (vi) Development of a multi-component dissemination and implementation strategy.

## Future Outlook

Over the upcoming months, work will focus on (i) completing the Center’s first 12 guidelines; (ii) approving the guidelines by the National Center for Evidence-Based Medicine’s Scientific Committee; (iii) disseminating the guidelines via multiple channels including a website, mobile apps, and educational events; (iv) implementing national guideline recommendations at the point of care through localized order sets embedded in the electronic health record at selected pilot sites; (v) finalizing the Center’s internal team and infrastructure; (vi) disseminating guidance on the GIN criteria for giving a seal of approval as national guidelines to those developed by other guideline organizations [7]; (vii) developing certified learning modules for a national training program for guideline development; (viii) fostering partnerships and collaborations with key stakeholders in the guideline ecosystem within Saudi Arabia as well as across the Middle East and worldwide; and (ix) establishing its strategic objectives for the upcoming years. This will enable the National Guidelines Center to realize its ambitious plans for extensive topic coverage, to take its place among the leading regional and global guideline developing organizations, and to optimally support clinicians and patients, Saudi Arabia’s healthcare transformation strategy, and the work of guideline communities worldwide.

## Data Availability

Not applicable.

## Abbreviations

VRPs: Vision Realization Programs
KSA: Saudi Arabia
GCC: Gulf Cooperation Council Countries
SHC: Saudi Health Council
HHC: Health Holding Company
RACI: Responsible/Accountable/Consulted/Informed
COI: Conflicts of Interest
EBM: Evidence-Based Medicine
GIN: Guidelines International Network (GIN)
GRADE: Grading of Recommendations Assessment, Development and Evaluation
